# On Acclimation

**Published:** 1850-09

**Authors:** Philip C. Williams

**Affiliations:** Winchester, Virginia


### THE MEDICAL EXAMINER,

AND

### RECORD OF MEDICAL SCIENCE.

NEW SERIES.—NO. LXIX.—SEPTEMBER, 1850.

### ORIGINAL COMMUNICATIONS.

On Acclimation. *An Inaugural Essay presented for the Degree of Doctor of Medicine in the University of Pennsylvania.* By Philip C. Williams, M. D., of Winchester, Virginia.

[Coucluded from page 450.]

As already intimated, there are great differences, both in an individual and national point of view, in the readiness of acclimation and the susceptibility to disease in a new climate.

It is a matter of common observation, says Levy, (*op. cit.* p. 528) that Northerners, going to the West Indies, suffer from yellow fever by the direct influence of an elevated temperature. He also states that delicate persons of lymphatic temperament have less to fear in a hot climate, than robust, energetic constitutions.

Another cause of mortality, so graphically described by the same author, is the dread or panic that takes possession of their minds, rendering them an easy prey to the violence of the epidemic.

But can none of these disastrous results be avoided? Experience shows that a strict observance of the following simple hygienic rules, may greatly mitigate, if not entirely prevent many of the diseases to which immigrants are now subject.

Bearing in mind the strong predisposition to inflammatory diseases exhibited by strangers in a hot climate, we readily see the importance of avoiding every thing that, in the least, tends to increase this predisposition. Hence it becomes of the highest importance to restrain the activity of the digestive and respiratory functions. With this view, we must begin as early as practicable, to change our mode of living, and endeavor to accustom our systems to the diet and habits which we must eventually adopt in order to secure happiness and comfort. Persons about to go to a hot climate, should commence this restriction in diet, &c., even before leaving home; and by all means should they exercise the strictest caution during their voyage; for it has been ascertained that the health of such persons is very materially affected by their habits during this period. (Johnson *op. cit.*) Having arrived at their destined port, they should bear up against those feelings of dread and alarm of which I have spoken; at the same time taking care not to fall into the opposite extreme of rashness and imprudence. They should be cautious and temperate both in food and drink. The *diet* should consist principally of vegetables, conjoined with a *small quantity of meat.*

Owing to the very excited and irritable condition of the intestinal canal, the use of stimulating food, or of condiments of any kind, should be avoided; so, also, should the new comers refrain from incautious indulgence in fruits, for to this cause has been attributed many of the diseases, particularly the cholera-morbus, of these countries. This danger is greatly increased by the free use of fruits in the evening. This remark applies with equal force to suppers, or to any unusual article of food taken at this hour.

The drinks should consist either of pure or slightly acidulated water, of water mixed with milk, or pure milk, when it agrees with the person using it. Buttermilk is preferred by some. Currie and Curtis recommend the use of stimulating drinks, pushed even to slight intoxication. There need, I think, be but little hesitation in condemning this practice. If such drinks be injurious when used in cold climates, how much more so must they be, when acting upon a system already overstimulated by a hot climate? I am satisfied that many of the disasters formerly attending migration to hot climates, can be traced to this unfortunate practice.

As this belief is opposed to the teaching of some, and the practice of many more, it may be well to examine the opinions of medical men who have had the most ample opportunities of testing the effects of alcoholic drinks, as a part of tropical hygiene. Johnson in his "Tropical Hygiene," says to those who go to hot climates: "the nearer we approach to a perfectly aqueous regimen in drink, during the first year at least, so much the better chance have we of avoiding sickness, and the more slowly and gradually we deviate from this afterwards, so much the more retentive will we be of that invaluable blessing—health." The celebrated Dr. Robert Jackson, physician general to the British army, thus speaks of himself: "I have wandered a good deal about the world, and never followed prescribed rules in any thing; my health has been tried in all ways; and by the aid of temperance and hard work, I have worn out two armies in two wars, and probably could wear out another, before my period of old age arrives. I eat no animal food, drink no wine, nor malt liquors, nor ardent spirits of any kind."

Dr. Mosely (on Tropical diseases,) says, "I aver from my own knowledge and custom, as well as from the custom and observation of others, that they who drink nothing but water, or make it their principal drink, are but little affected by the climate and can undergo the greatest amount of fatigue without inconvenience." "The hardy Arabs of the desert have no habitual drink but water. (Journal of Health, Vol. 2, p. 49.)

Dr. Marshall, the Deputy Inspector of Hospitals, observes, "I have myself marched on foot with troops on actual service, in a tropical climate whose mean temperature was considerably higher than that of Jamaica, without any other beverage than water and occasionally a cup of coffee. So far from being calculated to assist the human body in enduring fatigue, I have always found that the strongest liquors are the most enervating, and this in whatever quantity they are consumed." (*Op. cit.* p. 241.) Were it necessary the argument might be still further strengthened by other equally cogent examples; but enough has been said to show the deleterious effects of the use of ardent spirits as a part of tropical, and may we not say of any kind of hygiene?

We do not, on the other hand, mean to recommend the copious and indiscriminate use of very cold water. This drink, so refreshing in small quantities, is attended with considerable risk if the person have been enfeebled by prolonged exercise or by disease. On this account it should be used moderately in the latter part of the day. Cold or even iced water, in limited quantity, is both reviving and salutary, in the heat of the day, when the system is *moderately* excited. Its refreshing coolness relieves, or tends to diminish the heat of the stomach, and in this way somewhat abates the feeling of languor and debility which are the constant attendants upon protracted heat. On these accounts we hail with delight the introduction of ice as an article of commerce, thus conferring so great a boon upon the inhabitants of southern latitudes.

Of *Dress.*—It is desirable to lay aside linen and put on cotton garments, as the latter afford a much better protection against the vicissitudes of temperature, to which all hot climates are liable. If, as is frequently the case, the temperature at night be much lower than during the day, it would be advisable to avoid exposure at this time, or make some addition to the clothing. Persons obliged to labor in the open air and who perspire freely, will find it safer if not indispensably requisite, to wear next the skin, light flannel garments, which should be frequently changed, and well aired and sunned before being again put on.

There are many well authenticated proofs of the efficacy of this kind of clothing, in protecting its wearers from the deleterious effects of hot climates under which many have sunk who were not thus provided for. An interesting example to this effect is related by Dr. Combe, in his "Principles of Physiology." Capt. Murray, of the British man-of-war Valorous, preserved the health of his crew, numbering one hundred and fifty men, during a cruize in which he visited every island of the West Indies and the ports of the Gulf of Mexico, by making his men wear flannel next the skin, and by keeping the "between decks dry by means of stoves."

Bathing daily in cold water is eminently useful, by guarding the system against vicissitudes of temperature, as well as by tending to restrain excessive perspiration. The best time for its use is upon rising, in the morning, or at noon, if the system have been moderately stimulated by exercise. Andral, however, highly recommends its use in the evening, as it may induce sleep; for, as already mentioned, many suffer extremely from wakefulness. It may be well to caution against its use at this time, if the system have been worn out by the fatigues of the day, as it is thereby rendered unable to react under the depressing influence of a cold bath. With this precaution, it may, no doubt, prove beneficial. If, however, the person's constitution be at all times too weak to endure the bath, he may advantageously resort to sponging the whole surface of the body with salt and water, and follow this by careful and thorough friction with a coarse towel.

We ought to recommend the observance of regular and early hours—that is to retire to bed, and to rise early. To sleep upon a mattress of moss, hair, or something similar, adds much to one's comfort.

These habits should be strictly observed for two or three years, after which the system generally becomes so modified as to bear, if not to require, a fuller and more nutritious diet. But, extreme caution should be exercised in making any change from the plain and simple diet and habits that were adopted during the first period of acclimation.

I have spoken in considerable detail of the effects and hygiene of Southern acclimation, though not more so, I trust, than the interest and importance of the subject demand.

Equally striking as these, are the effects of a northern latitude upon a southern constitution. We will therefore now, for a short time, examine northern acclimation.

We have seen that the effects of moderate cold are, indirectly, to give greater activity to the nutritive functions; we have seen that it increases the activity of the circulation and respiration; so that all of these may assist in the development of animal heat, sufficient to maintain the temperature requisite for the healthy action of the different parts of the economy. On the other hand, we have seen, that, in warm climates, the demand for the evolution of heat is very limited, owing to the high temperature of the surrounding atmosphere. Hence we find calorification modified accordingly.

We may readily imagine, that, if one changes a southern for a northern latitude, this process and its dependencies must undergo a change—must be rendered more active, and thus the lungs, in order to supply the requisite quantity of oxygen, become stimulated to greater activity. This undue continued stimulation must result injuriously upon so delicate an organ. Of this fact we have a familiar illustration in the frequent deaths occurring in our menageries, among the animals of tropical regions; and *post-mortem* examinations reveal pulmonary, and generally tuberculous diseases.

We see the same tendency exhibited by all strangers who visit cold climates, or even by the natives of those regions who have been for a few years living in a warmer climate. Europeans, for example, who have lived some time in southern latitudes, are, on their return home, prone to be carried off, either by pneumonia or phthisis, or by the chronic diseases which they had contracted being changed into the acute form. Many examples of this tendency to tuberculosis, are afforded in the negroes who live far removed from tropical climates. Pearson relates several instances in which boys sent from Sierra Leone to England to be educated had died, in two or three years, of phthisis.

Statistical reports show that, in Canada, the number of deaths among the negroes from consumption, is such that, were not their numbers increased by immigration, the whole population would disappear in a few years.

The modifications of climatic influence, depending upon race, are very interesting, but unfortunately generally overlooked. " We find, then, that the number of deaths among the inhabitants of New York, of African descent, is twice as numerous as that among the whites of the same city. In Philadelphia the proportion is about three to four.

"If next we take particular classes, represented by equal numbers of white and black men, as soldiers for instance; in the same place, subject to the same discipline, and supplied with the same rations, we shall discover that the black troops are, in terrible disproportion, the greatest sufferers from disease, and furnish the largest number of deaths. Still following M. Boudin we learn, that, in Southern Europe, as at Gibraltar, the mortality amongst the blacks is three times that of the white soldiers; both of these corps being strangers to the soil. This immense disproportion is owing to the predominance of diseases of the respiratory apparatus.

" The mortality from pulmonary diseases among the negro soldiers in the British foreign posts is as follows: (The proportion is for every thousand.)

" On the western coast of Africa, it is 6.5; in Honduras, 8; in the Bahamas, 9.7; in Jamaica, 10.5; in the Mauritius, 12.5; in the Wind-ward Islands, 16.5; in Gibraltar, 35.5." (Pennsylvania Journal of Prison Discipline, January, 1850, p. 111.*

[* Since writing the above our attention has been directed to an article in the *Southern Medical Journal*, November 1, 1849, by Dr. Pendleton, of Sparta, Georgia, in which the views we have advanced find confirmation. It is republished in Dr. Fenner's *Southern Medical Reports*. It will be perceived, says Dr. Pendleton, that the whites are more subject to diseases of the *primæ viæ* than the blacks, a fact easily accounted for by their different modes of living, both as to exercise and diet. But upon what etiological principle is the latter race so much more subject to pulmonary affections? A little further on he says: the blacks, notwithstanding their greater exposure, seem to be less liable than the whites to periodic fevers, and more readily recover.]

I have neither time nor space to pursue this interesting subject as far as inclination prompts. I trust, however, that enough has been said, to show that the effects of a northern climate upon a southern constitution, are of an injurious, if not of an absolutely destructive character; and we are compelled to admit that there is *no such thing as a complete northern acclimation.*

In the South, if a person escapes for a few years, he may probably enjoy good health, at least sufficiently so to render life comfortable. But such is not the case in the North; for if the lungs be once seriously injured, the continuance of the cause must necessarily only increase the disease of these organs.

Much may, nevertheless, be accomplished, much suffering warded off, by a proper attention to dress, which should always be sufficiently thick to maintain the requisite temperature of the body. Flannel should be constantly worn next the skin, and the quantity of other clothing regulated according to circumstances, so as never to permit an abiding sense of chilliness.

The *food* should be as nutritious as possible, and should consist of animal and azotized substances, in order that the system may be supplied with a regular and abundant supply of fat, for this is found to be the most efficacious of all agents in the development and retention of animal heat. Proper limits are required even to this kind of food; and we should be careful to avoid all excesses, for this would defeat the object in view. Extreme care should be taken to keep the digestive and other functions in an active normal state. Not much is, I believe, to be affected by drinks. Some, however, recommend the use of tea, coffee, and even alcoholic liquors. The most that can be claimed for the last mentioned drinks, and particularly for distilled liquors, is the very moderate use of them. But actual experiment, both on sea and land, affords results by no means favorable even to this view. "Experience," says Dr. Bell, ('Baths and the Watery Regimen,' p. 189,) "has now corrected the once vulgar and prevalent opinion, that drinks more stimulating than water, and also of an intoxicating nature, such as ardent spirits, are necessary to enable men to bear great climatic extremes and vicissitudes. Dr. Miller, of New York, had, long before the temperance reformation, pointed out the instructive fact, that 'in all the frequent attempts to sustain the intense cold of winter in the Arctic regions, particularly in Hudson's bay, Greenland, and

Spitzbergen, those crews or companies which had been well supplied with provisions and liquors, and thus enabled to indulge in indolence and free drinking, have generally perished; while at the same time the greatest number of survivors have been uniformly found among those, who were accidentally thrown upon inhospitable shores, destitute of food and spirituous liquors, compelled to maintain an incessant struggle against the vigor of the climate, in procuring food, and obliged to use water alone for drink.'" These facts are too decisive to need any comment.

Cold bathing, if properly borne by the system, will be found very serviceable, but still more so will be sponging the skin with salt and water, and afterwards subjecting it to thorough friction with a coarse towel.

On the subject of hygiene, national habits merit our attention, if not our imitation. With a winter in our Northern States almost as cold as that of Russia or Scandinavia, we ought not to lose sight of the precautions by which the people of those countries protect themselves, both by the arrangement of their houses and their clothing.

The regular use of the vapor bath deserves consideration and attention. On this point I refer to Dr. Bell, (*op. cit.*) One good effect, at least, from this practice, must be to excite the skin to a proper discharge of its functions, and thus relieve the lungs and kidneys—organs, which we have seen, are so severely and injuriously tasked in cold climates.

The state of the general health should be most carefully watched, and the slightest tendency to disease particularly of the lungs, promptly combated.

We next come to the consideration of acclimation as modified by *moisture.* This, embracing as it does, the interesting, but extensive and complicated question of miasm, is too vast a field to be, in this place, fully explored. There are, however, some general considerations that demand attention.

In a preceding portion of this essay, I exhibited the picture drawn by Hippocrates, of the modifying influence of moisture. This is two-fold, and subject to modifications, according as it is associated with cold or with heat. To both of these I will briefly allude. The lungs and kidneys, which as we have said are in a state of minimum activity in a warm climate, are severely tasked in a cold and moist one. This is not so much owing to the direct operation of cold, as to its indirect effects, manifested through the enfeebled and impeded functions of the skin, by which these organs—the lungs and kidneys—are forced to extraordinary efforts to eliminate effete matters from the body. Thus it is that we see so great a tendency to renal diseases, and to tuberculosis and scrofulosis, which is exhibited in cold, and even during the winter seasons of temperate climates. This condition is greatly aggravated by a combination of moisture with cold, and to their joint agency may be attributed a long list of anginose, tracheal and bronchial affections.

Equally deleterious, though in an opposite mode, is the union of moisture and heat. This is supposed to be the most favorable condition for the development of miasmata. But as this is becoming, more and more, a controverted point, I shall not pretend to discuss it here.

Whatever may be the decision of this point, we may be quite sure of one thing, viz. that a hot and moist atmosphere is peculiarly depressing in its operation upon the functions, and renders the system *extremely susceptible, even if it does not originate,* attacks of fever, or of hepatic and splenic diseases. These effects are most liable to be experienced during the night, in hot and marshy regions. Among many illustrations may be quoted the following, as related by Dr. Lind. In 1766 the Phœnix, ship of war, was returning from the coast of Guinea. The officers and ship's company were perfectly healthy, till they touched at the island of St. Thomas. Here nearly all of them went on shore. Sixteen of the number remained for several nights on the island. *Every one* of these contracted the disorder (fever), and thirteen of the sixteen died. The rest of the crew, consisting of two hundred and eighty men, went in parties of twenty or thirty on shore in the day, and rambled about the island, &c., but they returned to the ship at night; *and not one of these who so returned, suffered the slightest indisposition.* Exactly similar events occurred the following year, with the same ship, at the same place, where she lost eight out of ten men, who imprudently remained out all night on shore, while the rest of the crew, who after spending the greatest part of the day on shore, always returned to their vessel before night, continued in perfect health, (Watson's Practice p. 450, Am.

edit.) Did time allow, I might refer to other facts to enforce the same point; but the above are sufficiently conclusive.

Another peculiarity of these situations is to develope diseases to which persons were predisposed, but in whom they had hitherto lain dormant. The following affords a striking illustration. Twenty eight soldiers being exposed, at work, in a malarious district, were all taken sick; not with the same disease, but three with cholera morbus, five with dysentery, four with yellow fever, and the remaining sixteen with pernicious intermittent fever." (Johnson.) With this necessarily imperfect description of the effects of moisture, I must hasten on to the subject of protective hygiene.

Persons going to such climates should carefully avoid all exposure to night air; they should not go out early in the morning, nor remain out late in the evening; and they should only go out after the sun has risen sufficiently long to dissipate the fogs and mists and to rarefy the atmosphere. They should avoid all exposure to wet; and refrain from all excesses in food or drink. Their diet should be nutritious; their drinks simple and pure. If the water for drinking be impure, it should be purified and filtered before being used. They should attend most strictly to their general health; to personal cleanliness; and should avoid every thing tending to debilitate and exhaust the system. Some recommend that, for a while at least, the system be kept under the slight influence of some tonics, particularly bark, or, still better, quinine.

After having, for a time, observed these precautions, and having overcome the primary excitement incident to hot climates, a more liberal diet may be adopted, accompanied with the use of condiments, such as capsicum, ginger, &c. It will be safer to continue a vegetable diet, combined with a small proportion of meat, until the acclimation be, in great measure, completed. It is then that the colonists may imitate, with advantage, the customs and habits of the natives, and of those who are inured to the climate.

But, in addition to these precautions, there is another of vital importance, viz. the selection of a site, exposure, &c., of the dwelling. They who are attracted to these countries, merely for the sake of commerce, and whose stay is short, are not so much concerned with this; nor, on the other hand, are they generally able to exercise such a choice. They who intend to become residents should give this subject their most careful consideration.

They often have the choice of situation—whether a low or a rising ground, a valley or a hill, is to be the site of their future abode. In this case they should always select an elevated and dry position. If this cannot be obtained—if they are compelled to build upon a moist situation, near a river, or stream, or still worse, near a stagnant pool—they must endeavor to discover the prevalent directions of the winds, and so place and arrange their houses that the wind shall blow against that side of the house containing few or no windows. If there be windows on that side they should never be opened till the sun has risen, and dissipated the mists, fogs, &c.; after which they may be thrown open, so as to ventilate the house. They should endeavor to screen it from these winds, by an intervening hill or strips of woods. If foiled in all these endeavors, and even though they may obtain these advantages, without the desired result, they may with great benefit keep their houses always dry and warm, either by furnaces or other convenient methods. There are many well attested instances in which persons have maintained perfectly their own health and that of their families, by this means, in the most sickly regions. In this way persons have been enabled to enjoy good health, even in the "Pontine marshes," and the "Campagna di Roma," and in otherwise uninhabitable regions. The sleeping apartments should never be on the ground floor; they should always be well aired and ventilated. For a fuller and more satisfactory exposition of this important subject, see Levy *op. cit.* art. "*Des Habitations.*"

The last variety of acclimation that I shall notice, is that incident to situations considerably elevated above the level of the sea.

As before stated, this elevation gives rise to greatly accelerated respiration and circulation; to pulmonary congestions; to dyspnœa; and sometimes to hæmoptysis and other hemorrhages.

It would seem, from the description of their feelings, by Gay Lussac and others who have ascended to great heights in balloons, that many of the effects attributed by travellers to their elevation above the level of the sea, are due, more to the fatigues of the ascent and the benumbing effects of cold than to a rarefied atmosphere. It is stated by some author whose name I forget, (possibly Humboldt,) that if we remain perfectly still on these heights, there is little or no disturbance of the respiration, or circulation; but if exercise, particularly when at all violent, be taken, all the symptoms above mentioned are fully developed.

This seems to show that the accelerated respiration, &c. must be referred to some other cause than cold; for if this were the explanation, these symptoms ought to be most violent when the person is at rest!

When we consider that the blood requires a certain proportion of oxygen to become properly oxygenated; and that in these situations there is a diminution of the requisite quantity of oxygen, we can easily conceive the necessity for the increased acceleration of the respiration to supply the deficiency. Hence we may have little hesitation in ascribing many of these effects to the rarefaction of the air, as well as to the "benumbing influences of the cold."

This variety of climate is only of interest on account of the fact that phthisical patients were formerly advised to remove to such situations. When we bear in mind, however, the variable temperature, and the excessively cold, damp and violent winds, to which these situations are liable, we cannot fail to admit the injurious effects of residence on them. For persons going to such places, it is necessary that they should adopt the same hygienic and other recommendations, suited to cold climates generally.

Did time permit we might speak of the various climates met with in the different parts of the United States; and particularly might we note the peculiarities of the climate of our middle States, in which occur the two extremes of great cold in winter, and excessive heat in summer.

These conditions call for different and almost opposite hygienic precautions, of which many, may I not say most, are neglected. Necessity forces us to make the requisite changes in clothing during the extreme seasons; but in the regulation of our food we still fall far short of the demands of a wise hygiene.

During the period of infantile life, the process of acclimation must be undergone at each return of our extreme seasons. To these extremes and transitions is to be traced the melancholy frequency of deaths occurring among infants—during the winter, from diseases of the pulmonary organs; during the summer from diseases of the intestinal canal.

Most fortunate would it be for the welfare of infants, if the profession regarded, and the mother understood, the peculiarities of our climate and the laws of acclimation.

